# The effectiveness of a web-based Dutch parenting program to prevent overweight in children 9–13 years of age: study protocol for a two-armed cluster randomized controlled trial

**DOI:** 10.1186/s12889-015-1394-1

**Published:** 2015-02-14

**Authors:** Emilie L M Ruiter, Gerdine A J Fransen, Gerard R M Molleman, Koos van der Velden, Rutger C M E Engels

**Affiliations:** Academic Collaborative Centre AMPHI, Primary Health Care, ELG 117, Radboud University Medical Centre, P.O. Box 9101, 6500 HB Nijmegen, the Netherlands; Behavioral Science Institute, Radboud University, P.O. Box 9104, 6500 HE Nijmegen, the Netherlands; Trimbos Institute, P.O. Box 725, 3500 AS Utrecht, the Netherlands

**Keywords:** Children, Overweight, Prevention, Parenting, Parental involvement, E-learning, Internet, Cluster randomized controlled trial

## Abstract

**Background:**

Although parental support is an important component in overweight prevention programs for children, current programs pay remarkably little attention to the role of parenting. To close this gap, we developed a web-based parenting program for parents entitled “Making a healthy deal with your child”. This e-learning program can be incorporated into existing prevention programs, thereby improving these interventions by reinforcing the role of parenting and providing parents with practical tools for use in everyday situations in order to stimulate a healthy lifestyle. Here, we report the research design of a study to determine the effectiveness of our e-learning program.

**Methods/Design:**

The effectiveness of an e-learning program was studied in a two-armed cluster randomized controlled trial. Parents of children 9–13 years of age who live in the Nijmegen region, the Netherlands, and who participated in the existing school-based overweight prevention program “Scoring for Health” were invited to participate in this study. Our goal was to recruit 322 parent–child dyads. At the school grade level, parents were randomly assigned to either the intervention group (which received e-learning and a brochure) or the control group (which received only the brochure); the participants were stratified by ethnicity. Measurements were taken from both the parents and the children at baseline, and then 5 and 12 months after baseline. Primary outcomes included the child’s dietary and sedentary behavior, and level of physical activity. Secondary outcomes included general parenting style, specific parenting practices (e.g., set of rules, modeling, and monitoring), and parental self-efficacy.

**Discussion:**

We hypothesize that children of parents who follow the e-learning program will have a healthier diet, will be less sedentary, and will have a higher level of physical activity compared to the children in the control group. If the e-learning program is found to be effective, it can be incorporated into existing overweight prevention programs for children (e.g., “Scoring for Health”), as well as activities regarding Youth Health Care.

**Trial registration:**

Dutch Trial Register: NTR3938. Date of registration: April 7^th^, 2013.

## Background

The increasing prevalence of overweight and obese children is a major health concern in developed countries [1-3], particularly among low socio-economic status (SES) neighborhoods in the Netherlands, which include high numbers of families of Turkish and Moroccan descent [4-6]. According to the 2009 Fifth Dutch Growth Study, 14% of children 2–21 years of age are overweight, a nearly three-fold increase from 1980. Striking, 2% of children are considered obese [4]. The overall prevalence of overweight and obesity among Turkish and Moroccan children is 2–4 times higher than among Dutch children [5]. Addressing this problem is important for preventing weight-related problems that can develop in childhood and/or adolescence.

In addition to regular physical activity and a healthy diet, parenting styles and practices are key components of interventions designed to prevent overweight in children, and incorporating parenting within these interventions can greatly increase their effectiveness [7-10]. For example, parents should be involved in these interventions, and they should be supported in the following roles: *i*) helping facilitate a healthy lifestyle, *ii*) using specific parenting practices, and *iii*) learning general parenting practices [10]. Specific parenting practices include specific, goal-directed parental actions designed to influence the child’s behavior, and these practices include establishing rules, as well as modeling and monitoring dietary, sedentary, and physical behaviors. General parenting is the emotional climate in which parents raise their child [11] and include how parents communicate with their child; general parenting has been characterized using dimensions regarding the parent’s responsiveness and demanding nature [12,13].

A literature search by Snoek et al. [10]—together with the results of workshops held within the field of practice in phase 1 of the Consortium Integrated Approach Overweight (CIAO) [14]—revealed that relatively little attention is given to the role of parenting within interventions for preventing overweight among children. Although many professionals stress the importance of involving parents in these interventions, this is often difficult to achieve in practice [14].

To close this gap, we developed an e-learning parenting program called “Making a healthy deal with your child”. This program can be incorporated into existing interventions for preventing overweight in children. The purpose of this e-learning program is to improve existing interventions by *i*) strengthening both the general and specific parenting practices and *ii*) increasing the self-efficacy of parents of children 9–13 years of age. This goal can be achieved by reinforcing the roles of parenting, by involving parents in existing interventions, and by giving parents practical tools that they can use to encourage their children to develop a healthy diet, be less sedentary, and engage in regular physical activity. In addition, the program is designed to give parents the tools they need to handle everyday life conflicts regarding dietary, sedentary, and physically active behavior. In the e-learning program, neither group sessions nor individual sessions must be followed; rather, the parents can follow the program in their own home, at a time that suits them best.

### Study aim

Here, we describe the study protocol and execution of a cluster randomized controlled trial (RCT) designed to investigate the effects of our web-based parenting program entitled “Making a healthy deal with your child” on dietary, sedentary, and physically active behavior among children 9–13 years of age who participate in the existing school-based overweight prevention program entitled “Scoring for Health”.

We hypothesized that 5 and 12 months after baseline measurements were collected, the children of parents who received the e-learning program would *i*) have a healthier diet (e.g., they eat more vegetables and fruits, have breakfast more often, and drink fewer sweetened beverages); *ii*) be less sedentary (e.g., will engage in a lower amount of screen-viewing time); and *iii*) have a higher level of physical activity compared to both their baseline values and the control group. Other objectives of the e-learning program include strengthening parenting styles, improving parenting practices, and increasing parental self-efficacy.

## Methods

### Design of the study

**Figure 1:**
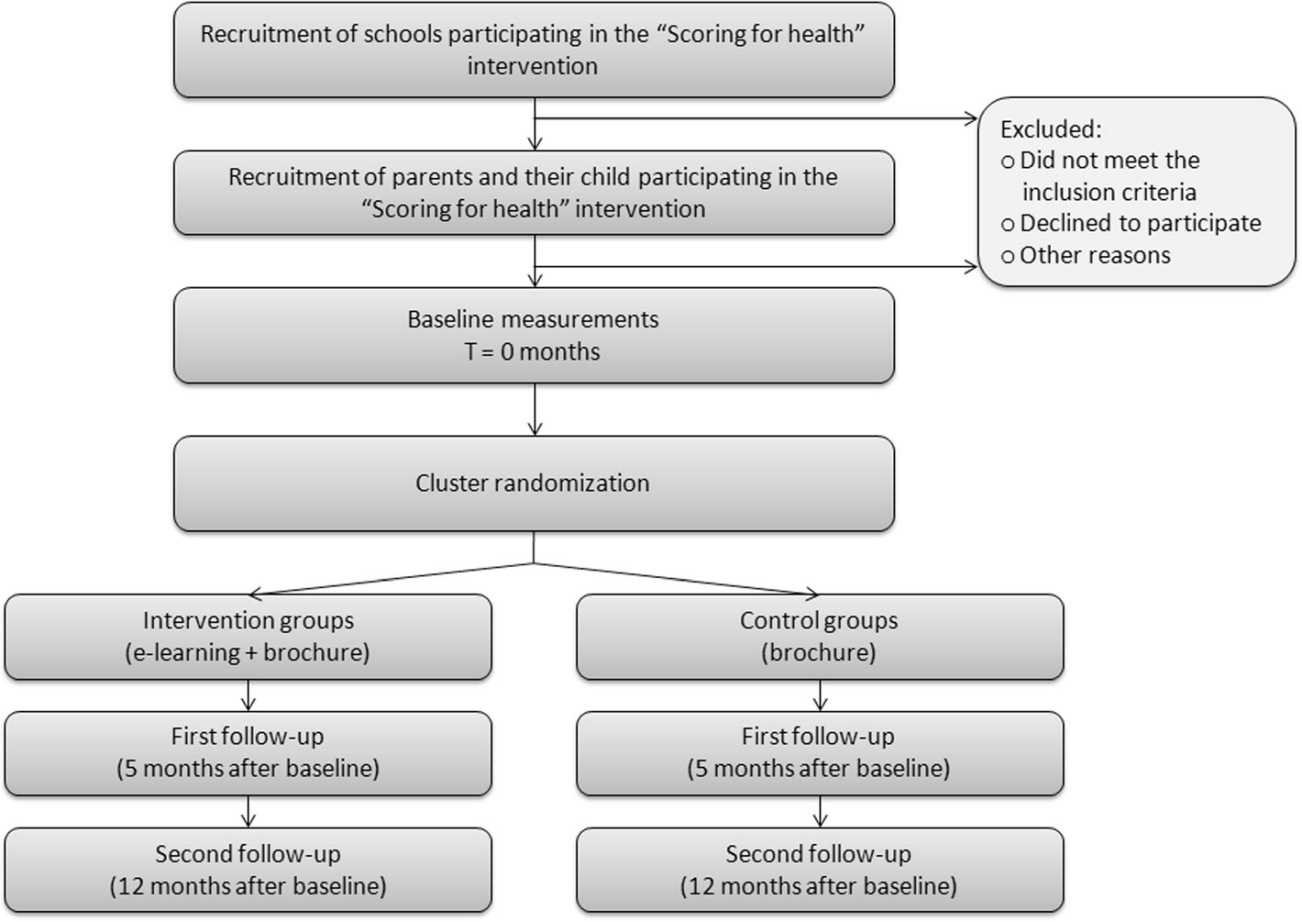
**Flow-chart of the parent–child dyads in the study.**

The effectiveness of this web-based parenting program was studied in a two-armed (intervention versus control) cluster RCT. The participants were parents and their 9-13-year-old children who were already participating in the existing school-based overweight prevention program entitled “Scoring for Health”. At the school grade level, the parents were randomly assigned to either the intervention group or the control group. Parents in the intervention group received a personal login code for the e-learning program and a standard brochure from the Nutrition Center regarding healthy eating and physical activity [15]; this brochure is also distributed by the organization Youth Health Care (YHC). Parents in the control group received only the brochure. Measurements were collected from the children and parents in both groups, as well as 5 and 12 months after the baseline measurements; see Figure 1.

In both groups, the parents received 30 Euros for their participation in the study. The children received a small bottle of water for their participation.

The Medical Review Ethics Committee of the Arnhem-Nijmegen region, the Netherlands, approved this study protocol, registration number 2012495, NL4280309112. This study did not require validation by the medical ethics committee. This trial is registered at the Dutch Trial Register (NTR3938).

### Participants and protocol

#### Inclusion criteria

Our inclusion criteria for recruiting parent–child dyads included parents in the Nijmegen region in the Netherlands and their children 9–13 years of age in the fourth, fifth, or seventh grade of regular primary school and who were participating in the existing school-based overweight prevention program entitled “Scoring for Health”. Parents and children were required to both speak and read Dutch. We selected “Scoring for Health” as an illustrative example, as it is a school-based intervention certified by the Center for Healthy Life (in Dutch, the *Centrum voor Gezond Leven*) in the Netherlands. Moreover, arrangements have been made to offer this intervention to a large group of children each year. Twice a year in the Nijmegen region, children 9–13 years of age are selected to participate in the “Scoring for Health” intervention program, yielding a total of 500 participating children.

The aim of the “Scoring for Health” program is to increase the awareness of primary school students (and their parents) regarding the importance of engaging in a healthy lifestyle. The program runs for 20 weeks and begins and ends with a sports clinic at a nearby semi-professional soccer club.

#### Recruitment

Eleven primary schools in the Nijmegen region in the Netherlands were participating in the intervention program “Scoring for Health”; the principals of these 11 schools were asked to participate in our study. We asked the principals’ permission to distribute envelopes to the parents of the children in the participating schools. The envelopes contained an invitation letter in which we asked parents to participate with their child in our study. In addition, the envelopes contained information regarding the study (including the purpose of the study, length of the study, frequency of measurements, eligibility criteria, confidentially of the data, etc.), a passive informed consent form for the parent, a passive informed consent form for the child, and an envelope for returning the forms. Two weeks after distributing the invitation letters, we visited all of the school classes. In our presence, the children completed the baseline questionnaire in class; the children were able to ask questions if they encountered ambiguities when completing the questionnaire. Thereafter, we distributed envelopes to the parents; this second envelope included the parental baseline questionnaire, a letter in which we asked the parents to complete the baseline questionnaire, and an envelope for returning the questionnaire. Non-responders received a reminder after three weeks. Our goal was to recruit 322 parent–child dyads.

#### Randomization

After baseline measurements were collected, an independent researcher at the Behavioral Science Institute randomly assigned all participating school classes to either the intervention group or the control group. Randomization was performed centrally at the school grade level class and within schools (to control for school characteristics) using a computerized random number generator with a blocked randomization scheme (block size was 2); the groups were stratified by ethnicity.

If more than one child living in the same household participated in the study, all participating children in that household were assigned to the same group as the oldest participating child in order to avoid contamination between groups.

#### Sample size

Sample size was calculated based on the difference between the intervention group and the control group in terms of the following outcomes: improvement in healthy eating and a reduction in sedentary behavior in children in accordance with Dutch standards. Because the e-learning program has not been tested previously with respect to effectiveness, it is difficult to estimate an effect size. However, based on the outcomes of similar effectiveness studies and school-based programs in children 9–13 years of age [16-19], we expect a minimum difference of 20% between the control and intervention groups 12 months after baseline in the children’s dietary and/or sedentary behavior in accordance with the Dutch standards.

Based on data obtained from the Community Health Service in Nijmegen, 40% of children 9–13 years of age meet the daily standards for eating fruits and vegetables, minimizing sugar-sweetened beverages, playing outside or minimizing sedentary behavior [20]. Using a two-sided test with alpha = 0.05 and a power of 0.80, and taking into account the clustering of children within classes (with an estimated ICC of 0.05 and an average cluster size of 14), we calculated that we needed 161 children in each group (i.e., the intervention and control groups) in order to detect an increase of 20% in the number of children who meet the Dutch standard with respect to dietary or sedentary behavior, given an initial compliance rate of 40% (i.e., an increase from 40% to 60%).

### Intervention

#### Program theory

The e-learning program “Making a healthy deal with your child” is based on the existing e-learning program “Talking with your child” [21], which is based on state-of-the-art knowledge regarding effective substance use–specific parenting [22], and the face-to-face parent-training program “Parents and children talking together”, which is designed to support parents with adolescent children with respect to their communication and conflict resolution skills [23-26]. The contents of “Talking with your child” are based on theoretical insights obtained from “Parent effectiveness training” [27] and “Parent management training – the Oregon model” [28]. Both of these programs are designed to build family relationships that are characterized by *i*) acceptance and non-judgmental attitudes towards each other, and *ii*) genuineness and being honest with respect to expressing feelings.

Using a six-step model, our program shows parents how to improve their communication and problem-solving skills. We transformed the original four-step model [26] into a six-step model by dividing the first step of the original four-step model (“discussing the problem and focusing on attentive and active listening, combined with giving opinions in ways that are most efficient and least intrusive for the child”) into three steps. The first step of our six-step model is “selecting a good time to discuss the problem”. The second step is “discussing the problem, combined with giving opinions in ways that are most efficient and least intrusive for the child”. The third step is “focusing on attentive and active listening”. The fourth step is “thinking of possible solutions for arguments between the parents and their child”; in this step, emphasis is placed upon brainstorming and giving all family members the opportunity to come up with solutions without judging or criticizing each other’s ideas. The fifth and sixth steps are “deciding on a solution to the particular argument” and “evaluating the chosen solution”, respectively.

For the theoretical knowledge regarding dietary, sedentary, and physically active behaviors, we used the guidelines of the Dutch Nutrition Center [29] and the Dutch Norm for Healthy Physical Activity [30], which were also used in the YHC.

#### Program content and structure

The aim of our e-learning program entitled “Making a healthy deal with your child” is to strengthen the general and specific parenting styles and practices in existing interventions for preventing overweight in children. At the start of creating this e-learning program, a pilot study was conducted with four focus groups in order to gain more insight into—and to obtain specific examples of—difficult daily life situations in which mothers in low-SES neighborhoods experience difficulties encouraging healthy eating habits and physical activity in their school-age children. The mothers in these focus groups were of Dutch, Turkish, and Moroccan descent and had at least one child 8–13 years of age.

In the study group sessions, the five most commonly mentioned difficult situations were as follows: *i*) the daily struggle at the dining table (children do not want to eat their vegetables); *ii*) the child’s continuous desire for candy and snacks; *iii*) children having insufficient time in the morning to eat breakfast; *iv*) children spending an excessive amount of time using the computer and/or watching television; and *v*) children not wanting to turn off their computer game when asked to do so.

Another important finding of the pilot study was that mothers in low-SES neighborhoods indicated that they experience several difficulties with respect to parenting. The most commonly mentioned issues were attempting to establish rules and not being strict and/or consistent when rules are established. The results of this pilot study were used as the input for developing our e-learning program. Our e-learning program consists of five 30-minute episodes that are based on the five abovementioned difficult daily life situations. Each episode consists of video fragments in which “good” and “less good” ways of communicating with a child are shown, the six-step problem-solving model, practical and theoretical assignments, and feedback. Using this approach, parents receive tools that they can use to encourage their child to develop a healthy diet, to be less sedentary, and to be regularly physically active in everyday situations; these tools use both general and specific parenting and conflict-management approaches. Using this e-learning program, our goal was to create a short, flexible program that is both simple and attractive to parents from a variety of demographic backgrounds. Parents can follow the program in their own home, at a time that suits them best. The total time required to complete the e-learning program is approximately 150 minutes.

#### Program delivery

Each parent who was assigned to the intervention group received a personal login code, which they used to start the e-learning program. Over a course of ten weeks, the parents were allowed to complete the e-learning program at their own pace. After finishing each episode, the parents received an e-mail to thank them for finishing the episode and to encourage them to complete the next episode.

#### Outcomes and specific measurement variables

We measured the effect of the e-learning program on energy balance–related behaviors in the children and on the parenting skills and parental self-efficacy in the parents.

The following primary outcomes were measured: changes in the child’s dietary, sedentary, and physically active behaviors from baseline to the 5-month and 12-month follow-up visits. According to Dutch standards [15,29,30], healthy energy balance–related behaviors in children include *i*) eating breakfast daily; *ii*) eating at least two portions of fruit daily; *iii*) eating vegetables daily; *iv*) drinking less than two glasses of sugar-sweetened beverages daily; *v*) less than two hours of screen time (watching television and/or using the computer) each day; *vi*) playing outside for at least one hour each day; and *vii*) participating in an organized sport for ≥30 minutes at least twice a week.

The following secondary outcomes were measured: parental styles, parenting practices, and parental self-efficacy. In addition, we monitored the parents’ willingness to follow the e-learning program and the parents’ satisfaction with the e-learning program.

#### Questionnaire

Parents and children were asked to complete a baseline questionnaire and again at the end of the “Scoring for Health” intervention program (5 and 12 months after the baseline assessment). The questionnaires for the children were administered in their respective schools, and the parents completed their questionnaires at home. The questionnaires were developed using existing validated Dutch questionnaires (or questionnaires that were used in currently ongoing projects within the Netherlands if no validated questionnaire was available). The questionnaires were designed to assess the following variables:

- Socio-demographic characteristics of the child: gender, age, current grade in school, and ethnic background [31];
- Socio-demographics of the parents: gender, age, ethnic background, household and family composition, highest level of education, current work situation (i.e., hours of paid work per week), height and weight (self-reported), and their perception of their child’s weight status [31];
- The child’s dietary behavior (fruit and vegetable consumption, whether they eat breakfast, their snacking behavior, consumption of sugar-sweetened beverages), sedentary behavior (television watching and computer usage), and level of physical activity. These questions were included in the questionnaires for both the parents and children [31];
- General parenting style, including restrictiveness and nurturance [32,33];
- Parental feeding style: instrumental feeding, emotional feeding, control, and encouragement [34,35]. This was measured for both the parents and child.
- Parenting practices, including dealing with house rules (strict, flexible, or no rules) [31] and modeling [36] and monitoring [36] of eating habits, sedentary behavior, and physical activity;
- Parental self-efficacy, including satisfaction with one’s own efficacy and effectiveness at solving problems [37]; and
- Parental satisfaction with the e-learning program, which was measured by including process evaluation questions in the post-test questionnaire at 5 months and questions regarding changes in their management of difficult behavior in the 1-year questionnaire.

#### Electronic database

The parents’ login activity in the e-learning program was monitored during the intervention period. This allowed us to monitor which parents began the e-learning program (and which did not). This approach also provided information regarding which episodes the parents completed.

#### Anthropometry

Anthropometric characteristics of the children (height and weight) were measured at baseline at the start of the “Scoring for Health program using a calibrated scale and measuring tape of the YHC in accordance with established guidelines [38]. All measurements were performed by a YHC professional or trainee. The child’s height was measured (while barefoot) to the nearest 0.1 cm using a model 208 body meter (SECA, Hamburg, Germany). The child’s body weight was measured (while wearing only underwear and while barefoot) to the nearest 0.1 kg using a portable digital model 899 scale (SECA). Body mass index (BMI, measured in kg/m^2^) was calculated using the weight and height measurements. BMI data were used to study whether the child’s weight status affected the parents’ willingness to participate in our study and/or complete the e-learning program.

#### Statistical analysis

Descriptive analyses will be used to determine whether randomization resulted in a balanced distribution of key demographic variables (e.g., the child’s age, gender and grade in school, as well as the parents’ education level and ethnic background) and the baseline primary and secondary outcomes. Continuous variables will be presented as the mean ± SD, and categorical data will be presented as the percentage of respondents within each of the possible categories. Moreover, the variables that had differing distributions between the two groups will be entered as confounders in all models that test the effectiveness of the e-learning program. The effect of the e-learning program on changes in the child’s dietary behavior, sedentary behavior, and physical activity, as well as the parenting style, parenting practices, and parental self-efficacy, will be tested in accordance with the intention-to-treat principle and in a completers-only framework using Mplus [39]. Intention-to-treat means that all participants will be analyzed in the condition to which they were randomly assigned. Missing data will be handled by multiple imputation. A total of 50 datasets will be completed using multiple imputation. Mplus will read the 50 datasets using the TYPE = IMPUTATION option and will perform the desired analyses for each dataset. Mediating the parameter estimates will then aggregate the results of the 50 analyses. With respect to the completers-only analyses, only the participants with scores at all time points will be included. In both the intention-to-treat and the completers-only analyses, the effect of the intervention condition will be compared to the control condition. Because the data have a multilevel structure (i.e., individuals are “clustered” within school grades), the individual respondents may not necessarily be independent within each grade. To correct for the potential non-independence (complexity) of the data, the TYPE = COMPLEX procedure in Mplus will be used; this procedure corrects the standard errors of the parameter estimates for dependency, yielding unbiased estimates. Finally, the results will be reported in accordance with CONSORT (Consolidated Standards of Reporting Trials) [40,41].

#### Timeframe

The recruitment and inclusion of participants began at the end of 2012. The baseline data were collected from January 2013 through December 2013. After the baseline data were collected the participants were randomized in February 2013 and September 2013. For each parent–child dyad, the follow-up measurements were collected at fixed time points, 5 and 12 months after the baseline measurements (see Table 1). All data were continuously collected, entered. The data will be analyzed and the results will be reported after the completion of the final follow-up measurements collected 12 months after baseline.

**Table 1:** **Timeframe of the study measurements**

**Locations of the participated primary school in the Nijmegen Region**	**Baseline measurements**	**Randomization**	**Follow-up measurements**	**Follow-up measurements**
	**T = 0**		**T = 1**	**T = 2**
	**Questionnaire 1**		**Questionnaire 2**	**Questionnaire 3**
	**Height and weight of the child**			
**Druten and West Maas & Waal**	January – February 2013	February 2013	June – July 2013	January – March 2014
**Wijchen and Balgoij**	September – October 2013	October 2013	February – March 2014	September – December 2014

## Discussion

We describe the design of a two-armed cluster RCT to evaluate the effectiveness of our web-based parenting program entitled “Making a healthy deal with your child”. The aim of this e-learning program is to improve the child’s dietary, sedentary, and physical activity behaviors using specific parenting practices, improve parenting styles, and increase the self-efficacy of parents of children 9–13 years of age who participate in the existing school-based overweight intervention program “Scoring for Health” in the Netherlands. This trial was designed to test our hypothesis that children of parents who complete the e-learning program will *i*) have a healthier diet, *ii*) be less sedentary, and *iii*) have a higher level of physical activity compared to children of parents who did not complete the program.

### Strengths and limitations

This study was strengthened by its cluster RCT design, the relatively large sample size (322 parent–child dyads), and follow-up measurements collected at 5 and 12 months, which will enable us to analyze the short-term and long-term effects of the program. Furthermore, we will gain insight into which parents (including the socio-demographic characteristics and weight status of their child) began the e-learning program, and which parents did not. We also will gain insight into which episodes in the program the parents completed, and how much of each episode the parents completed. Armed with this knowledge, we will identify which parents are—and are not—reached using this intervention, and this information will help maximize the scope of the e-learning program. Strengths of the e-learning program is, the program is theory-driven and is based on difficult everyday life situations experienced by parents. Second, the program consists of multiple components (video fragments, a six-step problem-solving model, assignments, and feedback), which is important given that reports show that multi-component programs reveal more effects than single-component programs [42,43]. Third, because the e-learning program is web-based, parents can follow the program in their own home, in their own time, and at their own pace; moreover, parents are not obliged to engage in a complex, time-consuming program. Finally, with respect to the ability to generalize the study results, if our analysis shows that “Making a healthy deal with your child” is effective, this e-learning program can be easily incorporated into other intervention programs designed to prevent children from becoming overweight.

Despite its strengths, this study has some limitations. First, it is likely that more motivated parents completed the e-learning program, which may limit our ability to generalize our results to all parents. Second, in using a within-school design (as opposed to a between-school design), possible contamination effects may have occurred between the intervention and control groups. To minimize these effects, the parents in the intervention group received the same brochure as the parents in the control group, and only the parents in the intervention group received a personal login code in order to start the e-learning program. Third, with respect to measuring the effectiveness of the e-learning program, we did not focus on the child’s BMI. The primary purpose of the e-learning program is to change the child’s unhealthy diet, sedentary lifestyle, and low physical activity, resulting in healthier behaviors. Therefore, we focused on healthy energy balance–related behaviors rather than BMI. Finally, the information regarding the behaviors of the children and parents was based entirely on self-reporting by the children and parents, which could have led to over-reporting and/or under-reporting as a result of social desirability and/or recall bias. Thus, to minimize social desirability and optimize measurement validity, we ensured the full confidentiality (i.e., anonymity) of our participants. To minimize recall bias, the interval between the time period and the measurements was relatively short (i.e., participants were asked to recall events from the past month or week), which likely increased the self-reporting reliability.

### Implications for practice and conclusions

If our analysis reveals that our e-learning program is effective, it can be incorporated into existing intervention programs designed to prevent overweight and obesity in children 9–13 years of age living in the Netherlands. Our extensive collaboration with the Community Health Services in the Region (Gelderland) and national networks provides considerable potential for ensuring the effective dissemination of information, as well as the sufficiently large-scale, structural incorporation of the e-learning program in several interventions, including “Scoring for Health”, activities offered by the YHC, and the Dutch national school-based program entitled “The Healthy School”. Our e-learning program can be easily incorporated into these programs and can provide flexibility with respect to where, when, and how it is implemented. In addition, our study increases our knowledge regarding the factors that both contribute to and foster intervention effects, which could result in the further improvement of our e-learning program and other universally implemented programs for overweight prevention. Finally, the results obtained from this trial—which are expected in 2015—will be communicated to both scientists and health professionals.

## Abbreviations

BMI: Body mass index
CIAO: Consortium integrated approach overweight
RCT: Randomized controlled trial
SES: Socio-economic status
YHC: Youth Health Care
